# Preventative effects of resveratrol and estradiol on streptozotocin-induced diabetes in ovariectomized mice and the related mechanisms

**DOI:** 10.1371/journal.pone.0204499

**Published:** 2018-10-01

**Authors:** Yunxia Li, Jinbing Huang, Yuan Yan, Jingjing Liang, Qiankun Liang, Yanyu Lu, Li Zhao, Hongfang Li

**Affiliations:** 1 Department of Physiology, College of Basic Medicine, Lanzhou University, Lanzhou, Gansu Province, China; 2 Function Laboratory in College of Basic Medicine, Lanzhou University, Lanzhou, Gansu Province, China; 3 Key Lab of Preclinical Study for New Drugs of Gansu Province, Lanzhou, Gansu Province, China; Stellenbosch University, SOUTH AFRICA

## Abstract

Resveratrol, a non-flavonoid polyphenolic compound, is structurally and functionally similar to estrogen and has drawn great attention for its potentially beneficial effects on diabetes. However, it is not known whether it shares the same protective effect against diabetes as estrogen and the underlying mechanisms. The aim of the present study was to investigate the protective effects of phytoestrogen resveratrol and exogenous 17β-estradiol against streptozotocin (STZ)-induced type 1 diabetes. Female mice were ovariectomized (OVX) and chronically injected with different concentrations of resveratrol (0.1, 1 or 10 mg/kg) and 17β-estradiol (0.01, 0.1 or 1 mg/kg) subcutaneously for 4 weeks, and the levels of blood glucose, plasma insulin, plasma antioxidant capacity, the changes of pancreatic islet cells and the expressions of glucose transporter 4 (GLUT4), insulin receptor substrate 1 (IRS-1) and phosphorylation of extracellular signal-regulated kinase (p-ERK) were detected. Resveratrol and 17β-estradiol significantly inhibited the increase of the blood glucose level and the rise of plasma malondialdehyde in STZ-induced diabetic mice, improved the levels of plasma antioxidant capacity and plasma insulin, protected the pancreatic islet cells, and increased the expressions of GLUT4 and IRS-1, but decreased p-ERK expression in skeletal muscle and myocardial tissue. The results suggest that resveratrol or 17β-estradiol shows obvious protection against STZ-induced diabetes in OVX mice, the mechanisms probably involve their ameliorating antioxidant activities and islet function, promoting muscle glucose uptake and inhibiting the expression of p-ERK.

## Introduction

The incidence of diabetes mellitus (DM) has been rising increasingly, particularly in post-menopausal women [1, 2]. Animal experiments indicate that male animals have a higher probability of getting DM; in addition, castrated animals have a lower probability of developing DM [3, 4] and ovariectomized female animals have a higher incidence of the disease than the respective sham-operated controls [5]. Together, this indicates that reduced serum estrogen levels in post-menopause could induce insulin resistance (IR), which is causally associated with DM. It is well established that estrogen can increase insulin sensitivity and improve glucose metabolism [5]. Yoshihara et al [6] reported that maturation of both mouse neonatal β-cells and β-like cells derived from human induced pluripotent stem cells (iPSC) were promoted by estrogen-related receptor γ (ERRγ). However, there are mixed views on the clinical potential for estrogen replacement therapy in treating DM, particularly as long-term use increases the risk of breast and uterine cancer.

Resveratrol (trans-3,4',5-trihydroxystilbene), a non-flavonoid polyphenolic compound found in peanuts, grapes and mulberries, can behave as estrogens by binding to estrogen receptors and has been suggested to be linked to the anti-diabetic effect in DM [7]. Previous studies have revealed that resveratrol could reduce blood glucose levels in streptozotocin (STZ)-induced diabetic rats [8], in normal rats and mice [9, 10], in mice fed a high-fat diet and type 2 diabetic (T2D) db/db mice [11], and in humans [12]. In addition, resveratrol can also relieve insulin resistance and increase insulin secretion in some models [13–15]. However, it remains unknown whether improved glycemic control was the cause or consequence of improved β-cell function. Curiously, in a streptozotocin (STZ)-induced diabetic rat model and insulinopenic model, resveratrol could also reduce hyperglycemia and improve the insulin secretion respectively [13–15].

Type 1 diabetes (T1D) results from pancreatic *β*-cell autoimmune destruction which leads to absolute insulin deficiency and plasma glucose increase. Insulin exerts its hypoglycemic effect in target tissues by binding to insulin tyrosine kinase receptors, resulting in the recruitment and phosphorylation of insulin receptor substrate (IRS) proteins. The phosphorylation of IRS can transmit receptor stimulation to the downstream of PI3 kinase/AKT and mitogen-activated protein kinases/extracellular signal-regulated kinase (MAPK/ERK) which also trigger the translocation of vesicles containing glucose transporter 4 (GLUT4) from intracellular storage pools to the cell membrane and thereby increase the uptake of glucose into the cell [16]. It has been reported that high levels of ERK and phosphorylated ERK (p-ERK) are found in adipocytes from T2D patients [16] and in red gastrocnemius from T1D rats [17].

Resveratrol and 17β-estradiol have beneficial effects in rodent models of T2D [9, 18], in T2D patients [12, 19], and in untreated T1D-like rats [20, 21]. However, this latter condition does not reflect their real effect due to interference from endogenous estrogen. Thus, we used a STZ-induced T1D mouse model in which the effect of endogenous estrogen was removed by bilateral ovariectomy to mimic post-menopausal status. For further understanding of mechanisms, we investigated the influences of resveratrol and 17β-estradiol on fasting weight, plasma total antioxidant capacity (T-AOC), malondialdehyde (MDA), and fasting plasma insulin levels (FPI) and on pancreatic islet cell morphology; we also studied the expression of glucose transporter type 4 (GLUT4), insulin receptor substrate 1 (IRS-1), ERK and p-ERK in skeletal muscle and myocardial tissue.

## Materials and methods

### Animals

Adult female Kunming mice that were not pregnant and weighed 22–26 g, were supplied by The Laboratory Animal Center of Lanzhou University (Lanzhou University, Lanzhou, China). Animals were allowed free access to chow and water. The room temperature was maintained at 25°C, with a natural alternating day and night pattern.

### Diabetes mellitus induction and drug preparation

All mice were anaesthetized with pentobarbital sodium (50 mg/kg body weight, ip) and then ovariectomized. An incision was made along the abdominal mid-line, the ovaries were removed from both sides, and then the wound was sutured. After recovering for one week, all animals were injected intraperitoneally with STZ in 0.1 mmol/L sodium citrate buffer, pH 4.5 (Sigma Chemical Co., St. Louis, MO, USA) at a dose of 50 mg/kg/day for 5 consecutive days to induce pancreatic islet cell destruction and persistent hyperglycemia as described previously [22, 23]. Some of the ovariectomized mice were administered resveratrol or 17β-estradiol (both dissolved in sesame oil, Sigma Chemical Co.) subcutaneously on every other day for 4 weeks, meanwhile at the beginning 5 days, STZ was simultaneously injected at a dose of 50 mg/kg/day intraperitoneally. Blood glucose levels were measured from the caudal vein using a portable glucose measuring device (Roche) once a week. Mice with hyperglycemia (blood glucose levels ≥300 mg/dl) were defined as a diabetic model, as before [23].

All animal procedures carried out in this study were reviewed, approved, and supervised by the Institutional Animal Care and Use Committee of the Ethics Committee of Lanzhou University, China.

### Experimental design

The mice were randomly divided into eight groups: Ovariectomy group (OVX), Ovariectomy+intraperitoneal injection of STZ (OVX+STZ), Ovariectomy+intraperitoneal injection of STZ +subcutaneous injection of 0.1 mg/kg resveratrol (R0.1), Ovariectomy+intraperitoneal injection of STZ +subcutaneous injection of 1.0 mg/kg resveratrol (R1), Ovariectomy+intraperitoneal injection of STZ +subcutaneous injection of 10 mg/kg resveratrol (R10), Ovariectomy+intraperitoneal injection of STZ +subcutaneous injection of 0.01 mg/kg 17β-estradiol (E0.01), Ovariectomy+intraperitoneal injection of STZ +subcutaneous injection of 0.1 mg/kg 17β-estradiol (E0.1), Ovariectomy+intraperitoneal injection of STZ +subcutaneous injection of 1 mg/kg 17β-estradiol (E1), with eight animals per group (n = 8). Five weeks after surgery, all mice were sacrificed by cervical dislocation. Total antioxidative capacity (T-AOC) was determined by colourimetry; malondialdehyde (MDA) was determined using the TBA colourimetric method; fasting insulin levels were determined by radioimmunoassay.

### Analysis of insulin resistance

We used Homeostasis Model Assessment of IR (HOMA-IR) as a fasting insulin-resistant test and used HOMA/β-cells (HOMA/β) as an index of islet β-cells function. HOMA-IR and HOMA-%β are calculated as follows: HOMA-IR = (FPI × FPG) / 22.5; HOMA-%β = (20 × FPI) / (FPG—3.5). Here, FPI is the fasting plasma insulin concentration I(μI∪/ml) and FPG is fasting plasma glucose (mmol/L) (Table 1).

**Table 1:** The levels of T-AOC, MDA, FPI, HOMA-IR and HOMA-%β.

Groups	Glucose in urine	T-AOC (U/ml)	MDA (nmol/ml)	FPI (μI∪/ml)	HOMA-IR	HOMA-%β
OVX	100% negative	11.94±1.44	8.31±1.29	39.68±1.19	12.64±1.17	216.43±1.17
OVX+STZ	100% positive	5.91±0.78***	12.08±1.28***	24.72±0.96***	14.17±0.99	52.62±0.99***
R0.1	100% positive	4.38±0.55	12.40±1.75	25.27±1.25	13.99±1.27	56.43±1.27
R1	100% positive	5.17±0.91	11.16±1.38	25.90±1.88	14.64±1.43	56.17±1.43
R10	56% positive	9.43±0.91^##^	9.49±0.93^#^	31.9±2.28^###^	13.27±1.56	108.83±1.56^###^
E0.01	100% positive	4.93±0.92	12.96±1.05	25.14±1.79	14.45±1.60	53.29±1.60
E0.1	50% weak positive	11.43±1.93^###^	8.16±0.83^###^	33.49±1.75^###^	13.03±1.47	127.46±1.47^###^
E1	62.5% weak positive	7.71±0.86^#^	9.72±0.68^#^	27.11±1.60	11.12±1.27	94.62±1.27^##^

T-AOC: Total antioxidant capacity; MDA: Malondialdehyde; FPI: Fasting plasma insulin; HOMA-IR: Homeostasis model assessment of insulin resistance; HOMA-%β: Homeostasis model assessment of β cells. The serum levels of T-AOC, MDA and FPI in ovariectomized and streptozotocin-induced diabetic mice treated subcutaneously with 0.1 mg/kg resveratrol (R0.1), 1.0 mg/kg resveratrol (R1), 10.0 mg/kg resveratrol (R10), 0.01 mg/kg 17β-estradiol (E0.01), 0.1 mg/kg 17β-estradiol (E0.1) or 1.0 mg/kg 17β-estradiol (E1). Ovariectomy group (OVX) was used as a normal control, Ovariectomy group and intraperitoneally injection of STZ (OVX+STZ) was used as a model group. Compared to OVX, ***p<0.001. Compared to OVX+STZ, #p<0.05, ##p<0.01, ###p<0.001. (n = 8).

### Western blot analysis

Skeletal muscle and myocardial tissues were lysed in RIPA lysis buffer with 1mmol/L phenylmethylsulfonyl fluoride (Sigma Chemical Co.). Protein levels were determined using a BCA Protein Assay Kit (Solarbio, Beijing, China). An equal amount of protein from each sample was subjected to electrophoresis on 10% sodium dodecyl sulfate-polyacrylamide gels, and subsequently transferred onto polyvinylidene fluoride (PVDF) membranes. After blocking with 5% bovine serum albumin (BSA) in TBST, membranes were individually incubated overnight with the corresponding primary antibodies (ImmunoWay Biotechnology Company, Newark, DE, USA). Blots were incubated at room temperature for 1h with the secondary goat anti-rabbit antibodies (LI-COR Biosciences, Lincoln, NE, USA), and the blots were developed using the Odyssey detection system (LI-CORBiosciences). Target proteins were visualized and representative images were presented. Densitometric analysis was performed using ImageJ software [24].

### RT-PCR analysis

Total RNA was extracted from skeletal muscle and myocardial tissues using Trizol reagent (Invitrogen, Carlsbad, CA, USA) and converted into cDNA by reverse transcription according to the manufacturer’s manual. The cDNA was used as PCR template for amplification. The following primers were used for PCR amplification:

1. *β-actin* gene:
  Forward, 5′-CATCCGTAAAGACCTCTATGCCAAC-3′
  Reverse, 5′-ATGGAGCCACCGATCCACA-3′
2. *GLUT4*gene:
  Forward, 5′-GACGGACACTCCATCTGTTG-3′
  Reverse, 5′-GCCACGATGGAGACATAGC-3′

The β-actin PCR was performed with 28 cycles of 30 s at 95°C, 30 s at 57°C, and 45 s at 72°C, followed by 10 min at 72°C. The GLUT4 PCR was performed with 26 cycles of 30 s at 95°C, 30 s at 57°C and 45 s at 72°C, followed by10 min at 72°C. The PCR products were separated on 2% agarose gel, and imaged.

### Immunohistochemistry

Skeletal muscle and myocardial tissues were fixed in neutral 4% paraformaldehyde solution, embedded in paraffin blocks, and cut into 5 μm-thick sections. Tissue sections were deparaffinized and rehydrated by a routine procedure. Deparaffinized sections were inserted into 0.01 mol/L citrate buffer with microwave heating, a solution of 3% H_2_O_2_ and 5% goat serum respectively. The samples were then incubated with an anti-GLUT4 antibody (ImmunoWay Biotechnology Company) overnight at 4°C. After washing, the slides were incubated with a secondary biotinylated antibody for 20 min at room temperature. Visualization was achieved using a DAB detection kit (Solarbio, Beijing, China) followed by staining with Harris hematoxylin for 10 s, liquid differentiation, and a neutral gum sealing piece. Finally, the sections were dehydrated using a graded ethanol series and xylene, air dried at room temperature, and sealed with neutral gum. We selected three slides prepared from three mice respectively to analyze GLUT4 expression.

### Observation of sections

All histological and immunohistochemical sections were evaluated using a microscope (Nikon Inc.) or using BI2000 image analysis system (Taimeng Co. in Chengdu, China) equipped with a trinocular microscope (Olympus, America) and a digital camera (Nikon, Japan). The images of GLUT4 expression in different groups of mouse skeletal muscle and myocardium were observed, analyzed and captured under Low Power Field (×100) and High Power Field (×400). The number of pancreatic islet cells was counted at a final magnification of 100× and averaged from at least 3 representative parts in one section in a blinded fashion by 1 observer.

### Statistical analysis

All data were expressed as the average ± standard deviation. The statistical differences were determined by Student t-test for comparison of two groups and ANOVA for comparison of three or more groups with SPSS 21 software. *P*<0.05 was considered statistically significant.

## Results

### Effects of resveratrol and 17β-estradiol on fasting weight and plasma glucose

**Fig 1:**
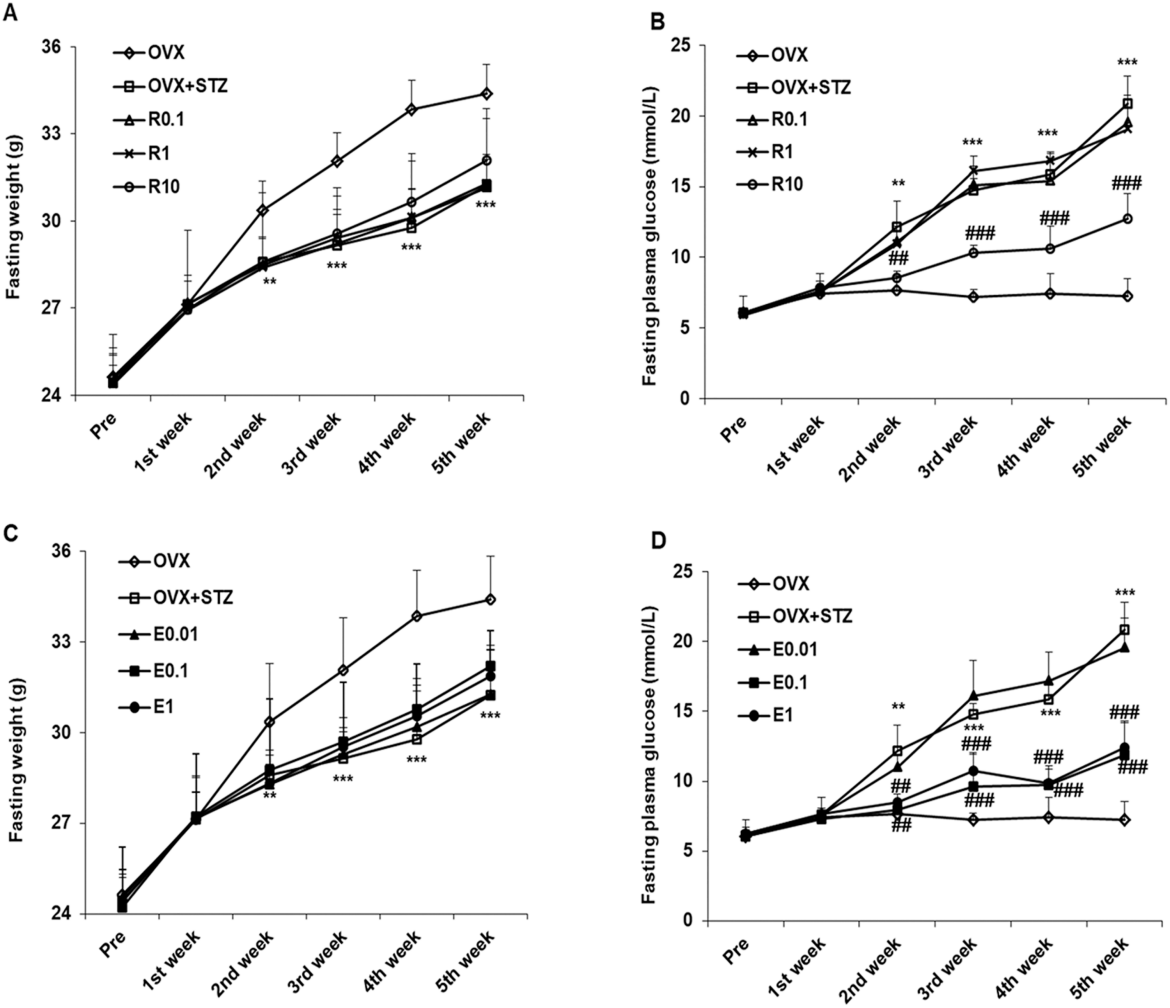
Changes of fasting weight and fasting plasma glucose. (A) Fasting weight and (B) Fasting plasma glucose in ovariectomized and streptozotocin-induced diabetic mice treated subcutaneously with 0.1 mg/kg resveratrol (R0.1), 1.0 mg/kg resveratrol (R1) or 10 mg/kg resveratrol (R10). (C) Fasting weight and (D) Fasting plasma glucose in ovariectomized and streptozotocin-induced diabetic mice treated subcutaneously with 0.01 mg/kg 17β-estradiol (E0.01), 0.1 mg/kg 17β-estradiol (E0.1) or 1.0 mg/kg 17β-estradiol (E1). Before operation (pre); the recovery period after operation (1^st^week); streptozotocin (STZ) was injected in the 2^nd^ week for 5 days, while resveratrol and 17β-estradiol were administered subcutaneously from the 2^nd^ week to the 5^th^ week for 4 weeks on alternate days. The ovariectomy group (OVX) was used as normal control, and the ovariectomy with an intraperitoneal injection of STZ group (OVX+STZ) was used as a model group. Compared to OVX, **p < 0.01, ***p < 0.001. Compared to OVX+STZ, ^##^p < 0.01, ^###^p < 0.001.

Fig 1A and 1C clearly demonstrate that in the ovariectomy group with an intraperitoneal injection of STZ (OVX+STZ), the mice fasting weight was significantly reduced when compared with the ovariectomy group (OVX) in the third, fourth and fifth weeks (p<0.001). No significant differences were found between different doses of resveratrol and 17β-estradiol treatment groups with the OVX+STZ group.

Fig 1B and 1D show that the OVX+STZ group had a significantly higher fasting plasma glucose level than the OVX group in the third, fourth and fifth weeks (p<0.001). Subcutaneous injection of 10 mg/kg resveratrol, 0.1 mg/kg17β-estradiol or 1 mg/kg 17β-estradiol led to a less increase in fasting plasma glucose than the OVX+STZ group in the third, fourth and fifth weeks (p<0.001). In contrast, subcutaneous injection of 0.1 or 1 mg/kg resveratrol, or 0.01 mg/kg 17β-estradiol (E0.01) did not lead to any significant difference in fasting plasma glucose compared to the OVX+STZ control.

### Effects of resveratrol and 17β-estradiol on serum T-AOC, MDA and FPI

Table 1 shows that T-AOC was reduced in the OVX+STZ group compared to the OVX group (p<0.001). After treatment with 10 mg/kg resveratrol, or 0.1 or 1 mg/kg 17β-estradiol, the T-AOC level increased significantly compared with the OVX+STZ group. The level of MDA was found to be significantly higher in the OVX+STZ group than the OVX group (p<0.001). Treatment with 10 mg/kg resveratrol, 0.1 or 1 mg/kg 17β-estradiol, led to a significant reduction in the level of MDA compared with the OVX+STZ group. The FPI level and HOMA-%β were reduced in the OVX+STZ group compared to the OVX group (p<0.001). After treatment, significant improvements were observed in the R10, E0.1 and E1 groups. Subcutaneous injection of 0.01 mg/kg 17β-estradiol, 0.1 or 1 mg/kg resveratrol had no significant effect on levels of T-AOC, MDA, FPI or HOMA-%β compared with the OVX+STZ control. HOMA-IR also showed no obvious changes in all groups.

### Effects of resveratrol and 17β-estradiol on pancreatic islet cells

**Fig 2:**
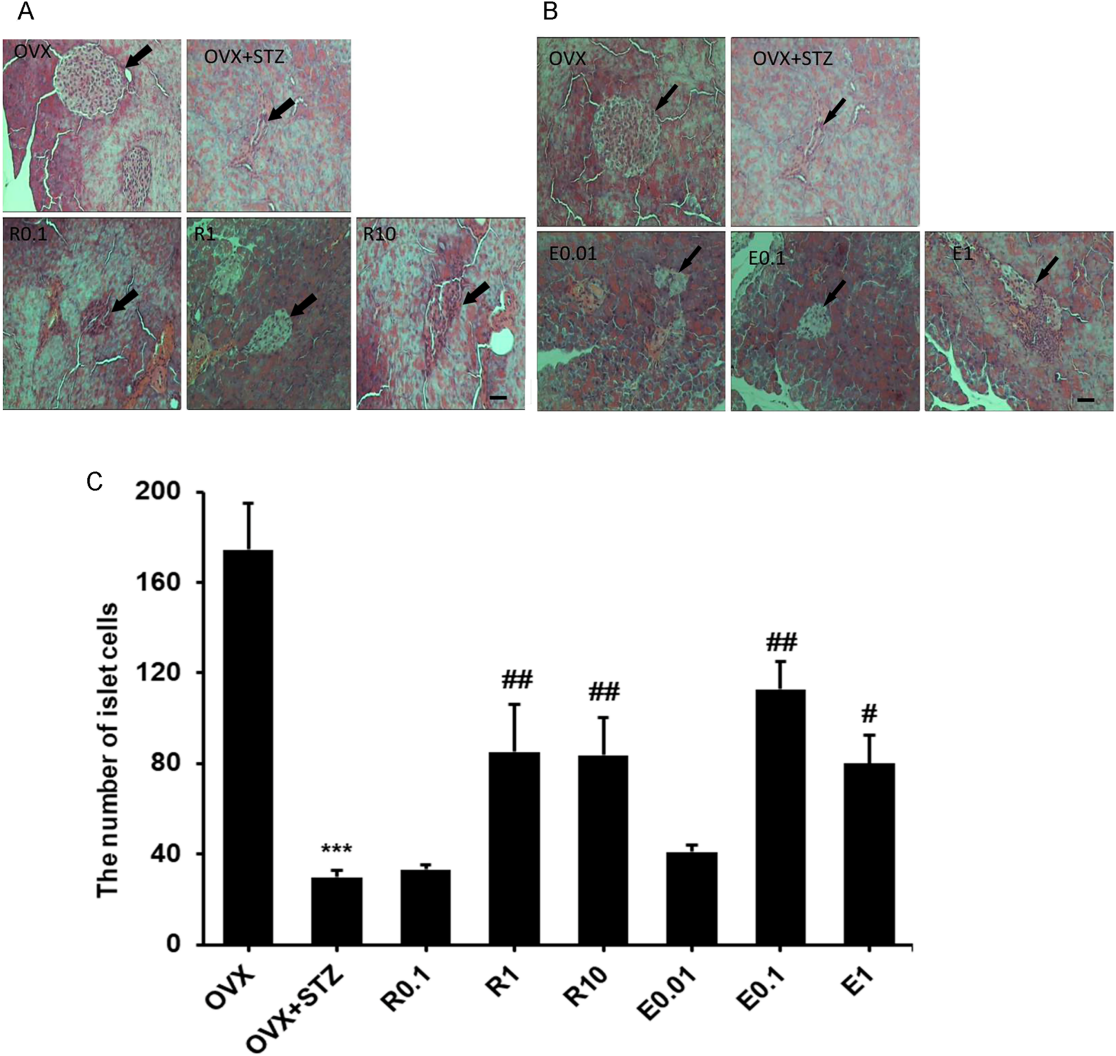
Changes of pancreatic islet cell morphology and numbers. Effects of resveratrol (A) and 17β-estradiol (B) on pancreatic islet cell morphology and numbers (C) in streptozotocin-induced diabetic mice treated subcutaneously with 0.1 mg/kg resveratrol (R0.1), 1.0 mg/kg resveratrol (R1), 10 mg/kg resveratrol (R10), 0.01 mg/kg 17β-estradiol (E0.01), 0.1 mg/kg 17β-estradiol (E0.1) or 1.0 mg/kg 17β-estradiol (E1) on alternate days. The ovariectomy group (OVX) was used as a normal control, and the ovariectomy with intraperitoneal injection of STZ group (OVX+STZ) was used as a model group. In figure (A) and (B): The scale bars are 20 μm. The arrows point to the islets. Compared to OVX, ***p < 0.001. Compared to OVX+STZ, ^#^p < 0.05, ^##^p < 0.01.

Compared with the OVX group, the pancreatic islet cells were observed to be smaller, their shape was deformed, and the number of cells was significantly reduced in the OVX+STZ group. After treatment with 1 or 10 mg/kg resveratrol and 0.1 or 1 mg/kg 17β-estradiol, the number of the islet cells increased significantly (Fig 2).

### Effects of resveratrol and estradiol on GLUT4, IRS-1 and p-ERK expression

**Fig 3:**
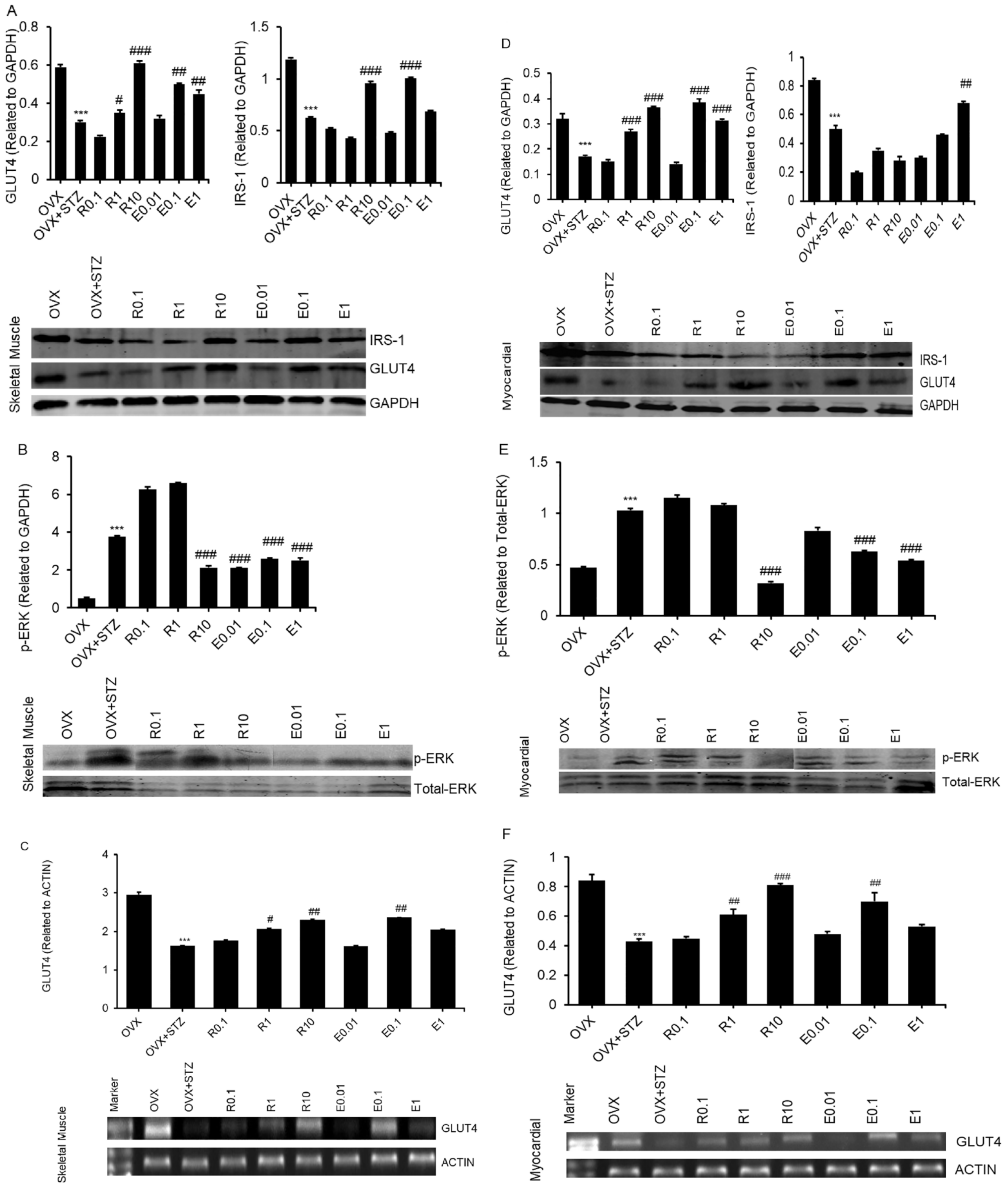
Effects of resveratrol and 17β-estradiol on GLUT4, IRS-1 and p-ERK/EKR protein expression in skeletal muscle and myocardial tissue. Western blot showing GLUT4, IRS-1 and p-ERK/EKR protein expression in skeletal muscle (A, B) and myocardial tissue (D, E) of ovariectomized and streptozotocin-induced diabetic mice treated subcutaneously with 0.1 mg/kg resveratrol (R0.1), 1.0 mg/kg resveratrol (R1), 10 mg/kg resveratrol (R10), 0.01 mg/kg 17β-estradiol (E0.01), 0.1 mg/kg 17β-estradiol (E0.1) or 1.0 mg/kg 17β-estradiol (E1). RT-PCR showing GLUT4 mRNA expression in skeletal muscle (C) and myocardial tissue (F). Data for western blot and PCR analysis was normalized to internal reference. GAPDH and ACTIN were used as internal references; the density values for GLUT4 and IRS-1 protein to GAPDH, p-ERK protein to ERK, GLUT4 mRNA to ACTIN were calculated, and the means ± S.E.M. for three different animals were shown. Compared to OVX, ***p < 0.001 (n = 3). Compared to OVX+STZ, ^#^p < 0.05, ^##^p < 0.01, ^###^p < 0.001(n = 3).

Compared with the OVX group, the expression of GLUT4 and IRS-1 in the OVX+STZ group were dramatically reduced, but the expression of p-ERK increased markedly in skeletal muscle (Fig 3A and 3B). After treatment with resveratrol and 17β-estradiol, the expression of GLUT4 and IRS-1 increased and the expression of p-ERK decreased significantly, especially in the 10 mg/kg resveratrol and 0.1 mg/kg 17β-estradiol groups (p<0.001) (Fig 3A and 3B). The expression of GLUT4 mRNA in skeletal muscle was similar to that of GLUT4 protein (Fig 3C). There were similar results observed in myocardial tissue (Fig 3D, 3E and 3F).

### GLUT4 protein expression detection by immunohistochemistry

**Fig 4:**
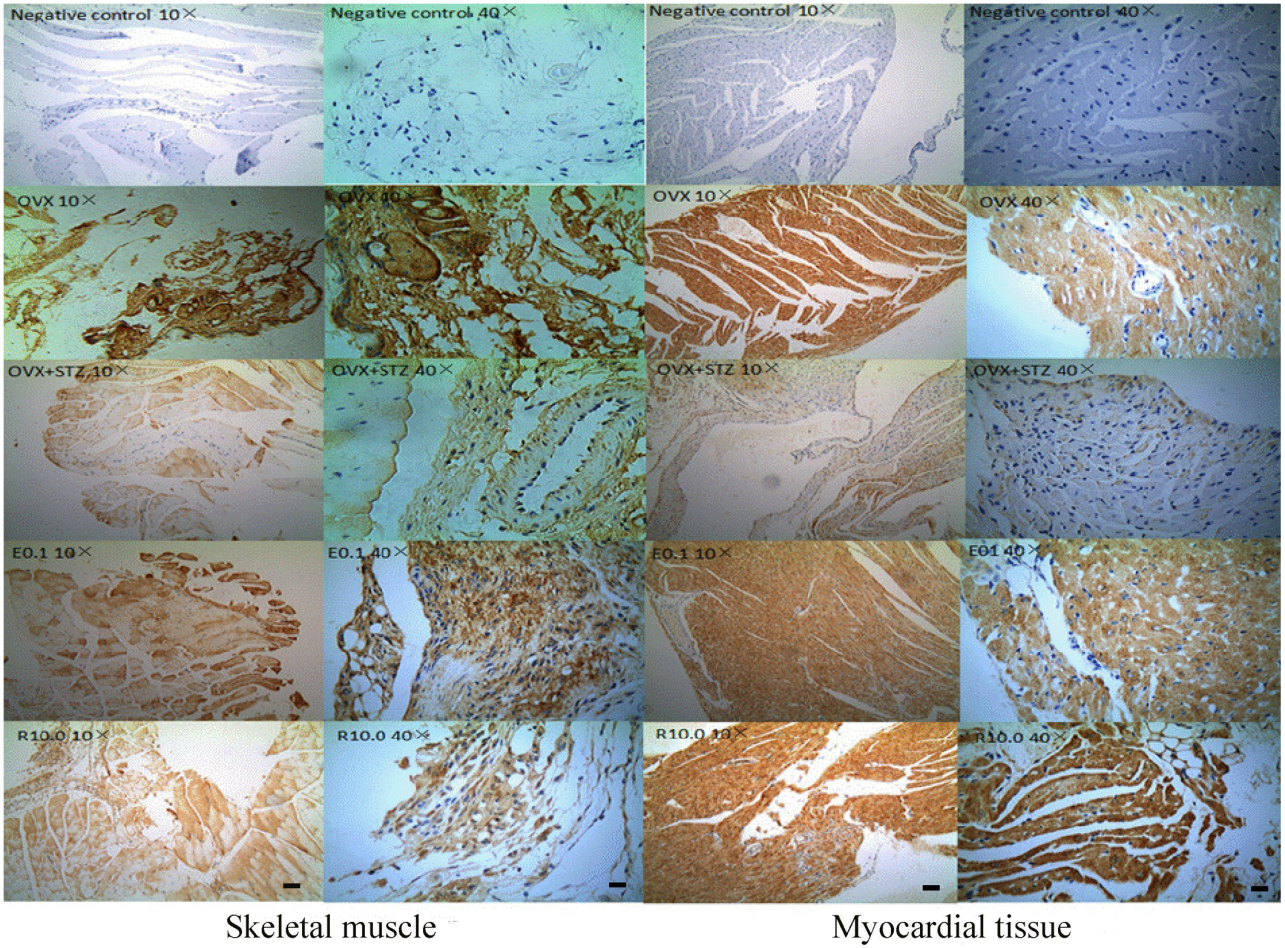
Immunohistochemical detection of GLUT4 expression in skeletal muscle and myocardial tissue. Skeletal muscle and myocardial tissue from ovariectomized and streptozotocin-induced diabetic mice treated subcutaneously with 10 mg/kg resveratrol (R10) and 0.1 mg/kg 17β-estradiol (E0.1). The ovariectomy group (OVX) was used as a normal control; the ovariectomy with an intraperitoneal injection of STZ group (OVX+STZ) was used as a model group. The scale bars are 20 μm in column 1 and 3, and are 5 μm in column 2 and 4.

Compared with the OVX group, the expression of GLUT4 was significantly reduced in both skeletal muscle and myocardial tissue in the OVX+STZ group. After treatment with resveratrol and 17β-estradiol the expression of GLUT4 increased significantly, especially in the 10 mg/kg resveratrol and 0.1 mg/kg 17β-estradiol groups (Fig 4).

## Discussion

The present experiment is the first to study the preventive and delaying effects of both resveratrol and estrogen on STZ-induced T1D in ovariectomized female mice. Although there are a few papers that reported the effects of resveratrol and estrogen on DM, most of them relate to T2D therapeutic effects which are the treatment of DM after successful modelling [10, 11, 13, 15, 25], and few of them focused on T1D. In order to investigate the preventive effects of resveratrol and exogenous estrogen on diabetes, a bilateral ovariectomy was performed to remove the effect of endogenous estrogen in the present study. Also ovariectomized animals are used as a model of post-menopausal females [26], their data will be very important and applicable in the prevention and treatment of post-menopausal diabetes, because epidemiological survey shows that DM incidence has been rising in post-menopausal women. In menopausal women who have impaired glucose tolerance, hormone replacement therapy can reduce blood glucose, increase insulin secretion and improve glucose tolerance [27–29]. Besides, some animal experiments have confirmed that female rats have reduced insulin sensitivity and glucose uptake after ovariectomy [30, 4, 5]. In our experiment, the induction of DM with STZ in ovariectomized mice led to the damage of mouse islet cells, thus obviously decreased the insulin secretion, which possibly caused some positive metabolic and inflammatory outcomes, such as the acceleration of the proteolysis and lipolysis, and therefore significantly increased the level of plasma glucose, caused glycosuria and reduced the body weight.

Resveratrol, a phytoestrogen, is a naturally occurring phenol compound which is structurally and functionally similar to estrogen [31]. Recently, data showed that orally administering resveratrol (40 mg/kg/day, a high dose) decreased albuminuria and attenuated the progression of kidney fibrosis in db/db mice [32, 33]. In contrast, a paper described that oral resveratrol at 0.5 mg/kg (a low dose) ameliorated DM symptoms and improved insulin resistance in a STZ-induced DM model [8]. In our experiment, resveratrol, similar to 17β-estradiol, could moderately, yet persistently inhibit the increase of blood glucose in ovariectomized mice induced by STZ over the experimental period of 4 weeks. Particularly, after subcutaneous administrated resveratrol at the dose of 10 mg/kg or 17β-estradiol at the dose of 0.1 mg/kg or 1 mg/kg, an obvious protection from hyperglycemia and a delaying onset of diabetes appeared in STZ-induced diabetic mice, but the doses of 0.1 mg/kg and 1 mg/kg resveratrol had no obvious anti-hyperglycemic effect, which probably involves the lower affinity of resveratrol combined with estrgenα-receptor (ERα) than that of estradiol. ERα is a key regulator in resveratrol- and estradiol-stimulating insulin-dependent and -independent glucose uptake and glucose homeostasis [34–36]. Why did the dose of 1.0 mg/kg17β-estradiol have less outspoken results than the dose of 0.1 mg/kg in the present study? The reasons are not completely understood. Because both physiological and genetic evidence indicate that estradiol and estrogen receptor are beneficial to the insulin sensitivity of rodents and humans when estradiol concentrations are closely maintained within the physiological concentration range, but super-physiological levels of estradiol or over-stimulation of estrogen receptors can induce hepatic insulin resistance and lower expression of muscle glucose transport protein [37, 38]. HOMA-IR has proved to be a robust tool for the assessment of IR as it is a model of the relationship of glucose and insulin, that predicts fasting steady-state glucose and insulin concentrations, while the HOMA-IR and HOMA-%β are indices of hepatic insulin resistance and β-cell function [39, 40]. However, in the present study, there was no apparent insulin resistance, because HOMA-IR showed no obvious changes in any of the groups. The dose of 0.1 mg/kg 17β-estradiol could induce the level of blood estrogen to reach the physiological concentration range which probably is the suitable blood concentration for anti-diabetic actions.

In the different anti-diabetes animal experiments, there is an argument about the level of plasma insulin in modelling animals. We showed that the pancreatic islet cells were damaged and the insulin secretion was decreased markedly in STZ-induced diabetic mice while resveratrol and 17β-estradiol increased the number of islet cells and HOMA-%β, protected islet cells and improved plasma insulin levels. This is consistent with previously reported observations by Palsamy & Subramanian whose experiment indicated that the level of blood glucose decreased and the level of plasma insulin increased significantly in STZ-nicotinamide induced experimental diabetic rats after orally treated with resveratrol (5 mg/kg/day) for 30 days [41], but inconsistent with the result reported by Su et al whose data showed that oral administration of resveratrol (0.5 mg/kg/day) for 14 days did not affect the insulin levels in STZ-induced diabetic rats [8]. The reasons for the different experimental results are probably related to the methods of replicating model, the doses and the time of administration resveratrol. Our results suggest that the hypoglycemic activities of resveratrol and 17β-estradiol are related to the protection of pancreatic islet and the improvement of insulin secretion.

Hyperglycemia is known to cause lipid peroxidation, which is associated with an elevated serum MDA concentration. Through exerting antioxidant effects [15, 42–44] and acting as anti-inflammatory agents [45, 46], resveratrol and 17β-estradiol completely alleviated oxidative stress, because both of them improved T-AOC and caused a reduction in MDA levels. Palsamy & Subramanian [47] reported that administration of resveratrol increased the activity of antioxidant enzymes to induce a reduction in reactive oxygen species activity and the restoration of tissue vitamin C and E reserves. This was accompanied by the increased levels of a transcription factor Nrf2, which is involved in combating oxidative stress in diabetes and is typically suppressed in animal models of DM [48, 49].

The toxicity of STZ not only stimulates oxidative stress, damages the pancreatic islet β cells and markedly reduces insulin secretion, but also causes the low-grade inflammation of multiple organs such as skeletal muscle and liver, and activates MAPK [50]. In our experiment, the OVX+STZ group had significantly higher p-ERK expression than the OVX group, in both skeletal muscle and myocardial tissue. Additionally, the OVX+STZ group had a significantly higher fasting plasma glucose level than the OVX group, suggesting that glucose may activate the ERK1/2 pathway, which is consistent with previously reported studies [51–53]. Treatment with resveratrol and 17β-estradiol prevented STZ-induced hyperglycemia, inhibited MAPK/ERK signal pathway and stimulated insulin secretion. Thus, we suggest that resveratrol and 17β-estradiol prevent STZ-induced T1D in part possibly by the blockade of the MAPK-mediated inflammatory cascade.

The insulin signalling pathway and the glucose transport system are also linked to MAPK/ERK cascade [16]. Insulin binds to the insulin receptor and then stimulates IRS phosphorylation. IRS-1 plays a key role in insulin signal transduction, and GLUT4 is necessary for insulin-stimulated glucose uptake [16]. Data suggest that the activation of MAPK signalling pathway may suppress the phosphorylation of IRS and downregulate GLUT4 expression, resulting in reduced glucose transport [54]. In the present study, resveratrol and 17β-estradiol could down-regulate p-ERK expression to inhibit the activation of MAPK, and then up-regulate GLUT4 and IRS-1 expressions, therefore promoted the glucose uptake and exerted the hypoglycemic effect. Previous studies, such as Chi et al reported that resveratrol could attenuate the expression of hepatic phosphoenolpyruvate carboxykinase (PEPCK) and enhance muscular GLUT4 expression, which indicates that the mechanisms underlying the hypoglycemic effect of resveratrol may be concerned with insulin-dependent and insulin-independent pathways, and that PI3K-Akt signaling may take part in glucose absorption in skeletal muscle [55]. Because the transport of vesicles containing GLUT4, essential for insulin-stimulated glucose uptake from intracellular stores to the plasma membrane is triggered by activated PI3K, the absorption of glucose is enhanced in the cell [56–60]. A paper has proved these proteins are important for maintaining insulin sensitivity in transgenic mice. According to the reports, GLUT4 reduction induces systemic insulin resistance and eventually diabetes [56, 58]. After IRS-1 is knocked out, the mice have a reduced rate of insulin-stimulated glucose uptake and diminished translocation of GLUT4 to the plasma membrane [57, 59].

In conclusion, the present study reveals the preventive nature and delaying effect of resveratrol and 17β-estradiol on the onset of diabetes in ovariectomized mice induced by STZ and provides the evidence that the preventive effects possibly contribute to the protection of pancreatic islet β-cells and the increase of insulin secretion. The finding also establishes the possible association of antioxidant activities in the protective effects of resveratrol and 17β-estradiol on islet β-cell function. Moreover, resveratrol and 17β-estradiol can produce a hypoglycemic effect in STZ-induced diabetic mice via ERK signalling pathway inhibition, and therefore enhance GLUT4 and IRS-1 expressions, promoting glucose uptake into skeletal muscles and myocardium. The results suggest that resveratrol, similar to estradiol, possesses a potential preventive effect for diabetes, particularly for post-menopausal diabetic women. In addition, the present study is of environmental nutrition value, as the foods and fruits containing resveratrol may have a preventive effect against diabetes.
